# Angiogenesis: Future of pharmacological modulation

**DOI:** 10.4103/0253-7613.62395

**Published:** 2010-02

**Authors:** Manisha Bisht, D.C. Dhasmana, S.S. Bist

**Affiliations:** Department of Pharmacology, Himalayan Institute of Medical Sciences, Dehradun, Uttarakhand, India

**Keywords:** Angiogenesis, antiangiogenic drugs, vascular endothelial growth factor

## Abstract

Angiogenesis is a fundamental biological process that is regulated by a fine balance between pro- and antiangiogenic molecules, and is deranged in various diseases. Historically, angiogenesis was only implicated in few diseases, such as, cancer, arthritis, and psoriasis. However, in recent years, it has been increasingly evident that excessive, insufficient or abnormal angiogenesis contributes to the pathogenesis of many more disorders. Research in angiogenesis offers a potential to cure a variety of diseases such as Alzheimer's and AIDS. Modulation of angiogenesis may have an impact on diseases in the twenty-first century similar to that which the discovery of antibiotics had in the twentieth century.

The process of forming new blood vessels is known as angiogenesis. Blood vessel formation is of two types. Vasculogenesis is the generation of blood vessels from endothelial cell progenitors (hemangioblasts). It is responsible for the formation of the primary vasculature of the body during early embryonic development.[1] Angiogenesis is a complex, highly regulated process, involving the sprouting, splitting, and remodeling of the existing vessels. Physiologically angiogenesis occurs under tight regulation in the female reproductive system and during wound healing. Many pathological conditions such as ischemic tissue injury are also benefited by revascularization. On the other spectrum, excessive angiogenesis, may result in different diseases including cancer, atherosclerosis, rheumatoid arthritis, Crohn's disease, diabetes, psoriasis, endometriosis, and adiposity.[2] These diseases may benefit from the therapeutic inhibition of angiogenesis.[2]

## History

The term angiogenesis was coined in 1787 by Dr. John Hunter, a British surgeon.[3] Professor Judah Folkman laid the foundation of angiogenesis research, when he put forward the idea that the growth of a tumor depended on their blood supply.[3] It was postulated that the tumor secretes some diffusible substance that stimulates the growth of new capillaries. Soon the basic fibroblast growth factor (bFGF) was shown to be capable of inducing an angiogenic response *in vitro*. Subsequently, many other angiogenic growth factors have been isolated, including the vascular endothelial growth factor (VEGF), which is a major mediator in tumor angiogenesis. In recent years, the angiopoietins have emerged as important regulators of angiogenesis. Several naturally occurring angiogenic inhibitors have also been discovered, such as, interferon-a/b, thrombospondin-1, angiostatin, and endostatin.

## Angiogenic Process

The vascular network formation consists of multiple coordinated, sequential, and interdependent steps mediated by a wide range of angiogenic factors, including growth factors, chemokines, angiogenic enzymes, endothelial specific receptors, and adhesion molecules. The process of neovascularization is halted due to the downregulation of angiogenic factors or the increase of inhibitors in the local concentration. Thus, angiogenesis requires a finely balanced equilibrium between pro-angiogenic and antiangiogenic factors.

## Steps of the Angiogenic Process

**Figure 1:**
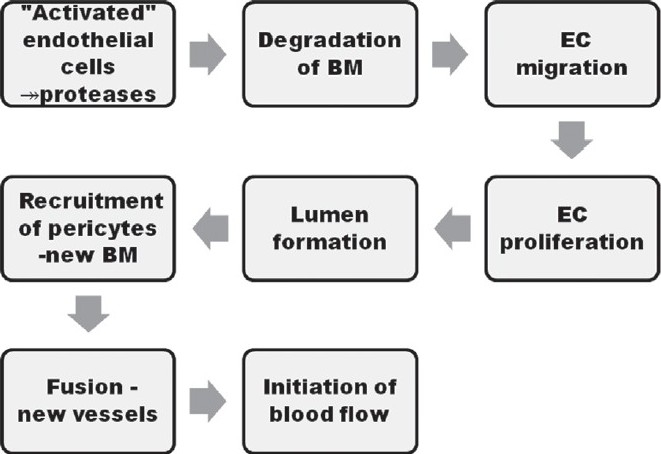
Steps of angiogenesis

The angiogenic process is divided broadly into three major steps including the initiation of the angiogenic response, endothelial cell (EC) migration, proliferation and tube formation, and finally the maturation of the neovasculature [Figure 1].

### 1. Initiation of the angiogenic response

Angiogenesis is initiated in response to hypoxia, by the release of hypoxia inducible factors (HIF), which facilitate the release of angiogenic stimulators, which in turn lead to EC activation. In both physiological and pathological angiogenesis, EC activation is the first process that takes place.[4] Activated EC secrete proteases, which degrade the extracellular tissue to facilitate endothelial penetration. Proteases may be broadly divided into matrix metalloproteases (MMPs) and the plasminogen activator (PA) / plasmin system. The MMPs are capable of degrading different protein types. PAs activate the plasminogen into plasmin, which degrades several components of extracellular matrix (ECM). Both PAs and MMPs are secreted together with their inhibitors, plasminogen activator inhibitors, and tissue inhibitors of metalloproteases, respectively, ensuring a stringent control of local proteolytic activity.

### 2. Endothelial cell migration, proliferation, and tube formation

Extracellular matrix degradation results in an increased concentration of various growth factors, which stimulate EC migration and proliferation. The ‘leader’ EC, followed by more EC, starts to migrate through the degraded matrix, thus forming small sprouts.[5] After the initial period of migration, rapid EC proliferation begins, thus increasing the rate of sprout elongation. These processes are also mediated by cell adhesion molecules.[6] Cell adhesion molecules can be classified into four families and neovascularization is facilitated by the member of each family.[6] Integrin α_v_β_3_and α_v_β_5,_ vascular endothelial cadherin, vascular cell adhesion molecule-1, P-selectin, and E-selectin are implicated in angiogenesis.

### 3. Maturation of the neovasculature

The final phase of the angiogenic process involves maturation of the neovasculature. The new basement membrane is synthesized by the newly forming capillaries. During this process extracellular proteolysis is locally inhibited to permit the deposition and assembly of the ECM components. After the formation of the capillary sprout, degradation of the newly formed ECM occurs again at the tip of the sprout, to allow further invasion. Interaction between the EC and ECM and the mesenchymal cells is a prerequisite for the formation of a stable vasculature. The polarity of the endothelial cells is established by cell adhesion molecules in order to form a lumen.[6] The platelet-derived growth factor (PDGF) regulates the recruitment of pericytes and smooth muscle cells required for further stabilization of the new capillaries. Finally, when sufficient neovascularization has occurred, the angiogenic factors are downregulated or the local concentration of the inhibitors increases. As a result, the endothelial cells become quiescent.

### Modulators of angiogenesis

**Figure 2:**
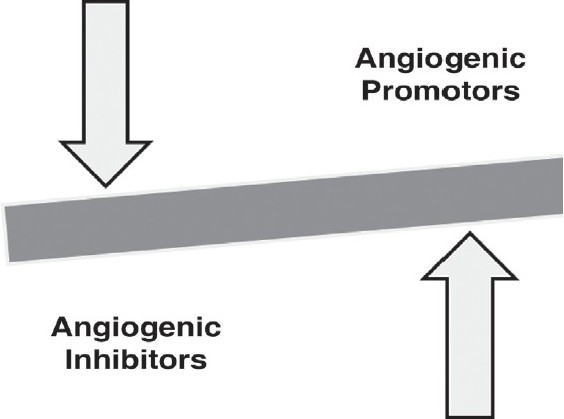
Angiogenesis: A dynamic balance between positive and negative regulators

Angiogenesis is a dynamic balance between the positive and negative regulators [Figure 2]. Both physiological and pathological angiogenesis are mediated by numerous ‘classic’ factors. Various angiogenic inhibitors have also been described, which inhibit the angiogenic process. Some of the factors have both pro- and antiangiogenic functions. A summary of the more important angiogenic activators and inhibitors is given in Table 1, although this list is by no means exhaustive.[7,8] Pharmacological manipulation of these factors may lead to the desired angiogenic response. Besides these, other mediators, such as, erythropoietin, angiotensin -II, endothelins, urotensin, leptin, adiponectin, resistin, neuropeptide Y, vasoactive intestinal peptide, and substance P were found to stimulate angiogenesis, whereas, somatostatin, ghrelin, and natriuretic peptides were found to inhibit it.[9] Of the long list of growth factors involved in the angiogenic process, VEGF, FGF-2, and angiopoietin-1 are considered to be the most important mediators of tumor angiogenesis. The characteristic of these factors is discussed here.

**Table 1:** Broad categorization of positive and negative regulators of angiogenesis[7,8]

*Positive regulators*	*Negative regulators*
Growth factors	Protein fragments
VEGF	Angiostatin
Placental growth factor	Endostatin
Fibroblast growth factors 1 and 2	Antiangiogenic antithrombin III
Pleiotrophin	Prolactin
Platelet derived growth factor	Soluble mediators
Transforming growth factor-α	Thrombospondin-1
Transforming growth factor-β	Troponin 1
Epidermal Growth Factor	Interferons
Insulin-like growth factor	Interleukins
Hepatocyte growth factor	Pigment epithelial derived factor
Angiopoietins	Platelet factor-4
Angiopoietin-1	Plasminogen activator inhibitor
Angiopoietin-2	Tissue inhibitors of metalloproteinaise
Cytokines and chemokines	Matrix derived
Tumor necrosis factor- α	Arrestin
Interleukin - 8 and 3	Canstatin
Prostaglandin E_1_, E_2_	Tumstatin
Enzymes	Fibulin
Thymidine phosphorylase	Endorepellin
Cycloxygenase-2	Other angiostatics
Angiogenin	2-methoxyestradiol
Cell adhesion molecules	PEX
Integrins	Vasostatin
Vascular cell adhesion molecule -1	
E - Selectin	
Vascular endothelial cadhedrin	
Miscellaneous	
Plasminogen activators	
Matrix metalloproteinase	
Plasminogen activator inhibitor	
Oestrogens	
Proliferin	
Leptin	
Erythropoetin	
Granulocyte colony stimulating factor	
Granulocyte-monocyte colony	
stimulating factor	

Nitric oxide

### VEGF (VEGF-A)

VEGF (VEGF-A) is one of the most potent proangiogenic factors. It belongs to the VEGF family, which includes other structurally related growth factors, such as, VEGF-B, VEGF-C, VEGF-D, VEGF-E, and PlGF.[10] VEGF is expressed in various tissues, including the brain, kidney, and liver.[10] VEGF stimulates most steps of angiogenesis and promotes angiogenesis. One of the most important inducer of VEGF is hypoxia.[11] Besides, many other growth factors including PDGF, EGF, TNF-α, TGF-β, and IL-1β mediate their angiogenic action by inducing VEGF expression.[10] VEGF mediates its action by an interaction with two VEGF-specific tyrosine kinase receptors, VEGFR-1 and VEGFR-2. VEGF signals mainly through VEGFR-2, whereas, the role of VEGFR-1 is not very clear in angiogenesis. VEGF plays a crucial role in physiological angiogenesis, as well as, has a role in several human cancers, diabetic retinopathy, rheumatoid arthritis, and atherosclerosis.

### Fibroblast growth factor (FGF-2)

The FGF family consists of nine structurally related polypeptides, all having a high affinity for heparin. FGF-2 was one of the first angiogenic factors to be characterized.[12] High concentrations of FGF-2 are found in the brain and pituitary gland. FGF-2 shows angiogenic activity *in vivo.*[12] The effects of FGFs are mediated via high-affinity tyrosine kinase receptors, of which at least four members have been described. FGF-2 is implicated in both physiological and pathological angiogenesis. FGF-2 is thought to play a role in the growth and neovascularization of solid tumors. High levels of FGF-2 are present in the EC of Kaposi's sarcoma and in the serum and urine of patients with many types of advanced cancinomas.[13]

### Angiopoietins

Angiopoietins are endogenously secreted glycoproteins that include angiopoietin-1 (Ang-1) and angiopoietin-2 (Ang-2). Both angiopoietins bind to Tie-2 (for tyrosine kinase with immunoglobulin and EGF-like domains), present on the endothelium, but only the binding of Ang-1 results in angiogenesis.[14] Therefore, Ang-2 is a natural antagonist of Ang-1.[15] VEGF and angiopoietins have complementary roles in angiogenesis.[16] During embryogenesis, VEGF promotes differentiation and proliferation of EC and the formation of immature vessels. Ang-1 induces the remodeling and stabilization of blood vessels, which involves interactions with the ECM.

### Models to study the angiogenic process

#### In vitro models

Various *in vitro* models are available for studying most of the steps in the angiogenic cascade, including EC proliferation, migration, and differentiation.[17] The proliferation studies are based on cell counting, thymidine incorporation or immunohistochemical staining. Cell migration can be studied by determining chemotaxis using the Boyden chamber.[17] Cells are added to the top well, chemotactic solutions are placed in the lower well, and after a period of incubation the cells that have migrated toward the chemotactic stimulus are counted. Differentiation can be induced *in vitro* by culturing EC in different ECM components, including fibrin clots, collagen gels, and matrigel. Advantages of *in vitro* models include the ability to study the individual steps of angiogenesis, the possibility to control the different parameters involved, and lower costs and effort. However, these models have inherent limitations that their effect may not be reciprocated in *in vivo* models. Therefore, the *in vitro* efficacy of a compound has to be substantiated by *in vivo* models.

### In vivo models

Chick chorioallantoic membrane (CAM), rabbit cornea assay, sponge implant models, matrigel plugs, and conventional tumor models are the classical *in vivo* angiogenesis models.[18]

**Chick chorioallantoic membrane assay:** The early chick embryo lacks a mature immune system and is therefore suitable for the study of tumor-induced angiogenesis. Tissue grafts are placed on the CAM and increase in vessel number and a typical radial rearrangement is seen. Blood vessels entering the graft are counted under a stereomicroscope. It is the most widely used assay for screening purposes as it is relatively simple and inexpensive. However, as the CAM already contains a well-developed vascular network, it is difficult to discriminate between new capillaries and already existing ones.

**Rabbit Cornea Assay:** This is very reliable method as the cornea presents an avascular site. Any vessel penetrating from the limbus into the corneal stroma is identified as newly formed. Slow release polymer pellets containing an angiogenic substance are implanted in ‘pockets’ created in the corneal stroma of the rabbit, to induce an angiogenic response. Computer image analysis after perfusion of the cornea with India ink is used to quantify the vascular response. This method is technically more demanding and more expensive.

**Sponge Implant Models:** Angiogenesis is frequently studied by the subcutaneous implantation of artificial sponges in animals. Sponge implantation is associated with non-specific immune responses, which produce significant angiogenic responses. An angiogenic response is detected either histologically, morphometrically (vascular density), biochemically (hemoglobin content) or by measuring the blood flow rate using a radioactive tracer. Direct data comparison becomes difficult due to the differences in sponge materials, shape, and size.

**Matrigel Plugs:** Matrigel is a matrix of a mouse basement membrane neoplasm. It is a complex mixture of basement membrane proteins and various growth factors. Subcutaneous injection of matrigel containing growth factors can be injected in mice. The matrigel plugs are removed after 10 days and angiogenesis is quantified histologically or morphometrically in the plug sections. Although it is expensive, matrigel provides a more natural environment compared to artificial sponges, to initiate an angiogenic response.

**Conventional Tumor Models:** Numerous animal tumor models like C6 rat glioma, B16BL6 melanoma, lewis lung carcinoma (LLC), and Walker 256 carcinoma have been developed to test the antiangiogenic and anti-cancer activity of potential drugs. Subcutaneous implantation of tumor cells is done and the tumor size is determined at regular time intervals. The efficacy of potential antiangiogenic agents can be evaluated on strongly vascularized tumors.

## Pathological Angiogenesis

### Insufficient angiogenesis

Both angiogenesis insufficiency as well as excess can lead to various disorders. Insufficient angiogenesis is characteristic of many disorders, including ischemic tissue injury or cardiac failure, where angiogenesis should be enhanced to improve the disease condition. The delayed healing of gastric ulcers has been attributed to the ability of the Helicobacter pylori to produce angiogenic inhibitors, whereas, reduced VEGF levels are responsible for recurrent aphthous ulcerations.[19] Organ dysfunction occurring in pre-eclampsia is associated with lower levels of VEGF.[20] Many age-related diseases such as nephropathy[21] and bone loss[22] have been found to be associated with progressive loss of the microvasculature. Age-dependent reductions in vessel density in the skin cause vessel fragility leading to the development of purpura, telangiectasia, palor, angioma, and venous lake formation.[23] Respiratory system disorders such as pulmonary fibrosis[24] and emphysema[25] are also associated with reduced angiogenic signaling. The extent of angiogenesis has also been correlated with survival in stroke patients[26] and insufficient VEGF levels are associated with motor neuron degeneration and are reminiscent of amyotrophic lateral sclerosis.[27] Amyloid-β, which has a role in the pathogenesis of Alzheimer's disease has adverse effects on the cerebral vasculature.[28]

## Excessive angiogenesis

Excessive vascular growth contributes to numerous disorders, and the list is growing rapidly. Cancer, arthritis, psoriasis, and blinding retinopathy are already known disorders associated with excess angiogenesis. However, numerous other disorders are characterized by excessive vessel growth. These include many inflammatory, allergic, infectious, traumatic, metabolic or hormonal diseases, such as, atherosclerosis, restenosis, transplant arteriopathy, warts, scar keloids, synovitis, osteomyelitis, asthma, nasal polyps, choroideal and intraocular disorders, retinopathy of prematurity, diabetic retinopathy, AIDS, endometriosis, and uterine bleeding.[29] In recent times, studies have shown that a high-fat diet induces an angiogenic gene program in fat tissues, which stimulates adipogenesis, while treatment of obese mice with antiangiogenic agents results in weight reduction.[30] Infectious diseases are also angiogenic. Viral and bacterial pathogens carry angiogenic genes or induce the expression of angiogenic genes in the host.[31] The human herpesvirus 8 transforms EC and causes Kaposi's sarcoma in HIV-1 infected AIDS patients. Similar to diabetic retinopathy, there is increased glomerular expression of vascular growth factors that contribute to diabetic nephropathy by promoting vessel leakage.[32]

## Therapeutic Angiogenesis (Induction of Angiogenesis)

Therapeutic angiogenesis would be beneficial in a variety of diseases. Table 2 tabulates a list of various conditions, which are likely to be benefited by the clinical manipulation of angiogenesis.[7] Becaplermin is an already approved topical recombinant PDGF preparation indicated for diabetic neuropathic lower extremity ulcers. Various angiogenic approaches to treat ischemic diseases are already in clinical trials.[33]

**Table 2:** Clinical modulation of angiogenesis[7]

*Inhibition of angiogenesis*	*Stimulation of angiogenesis*
Approved indications	Approved indications
Advanced cancer	Chronic wound - diabetic ulcer,
Ocular neovascularization	Experimental indications
Kaposi Sarcoma	Myocardial ischemia
Experimental indications	Peripheral ischemia
Hemangiomas	Cerebral ischemia
Psoriasis	Reconstructive surgery
Rheumatoid arthritis	Gastroduodenal ulcers
Endometriosis	
Atherosclerosis	

Adapted from Liekens et al. Angiogenesis: regulators and clinical applications[7]

Angiogenesis can be induced mainly by two methods. Either angiogenic proteins or endothelial progenitor cells synthesizing angiogenic growth factors can be injected directly into a site to stimulate blood vessel growth, or the right genes can be activated to induce a signaling cascade that would lead to angiogenesis. The delivery of VEGF or bFGF to the ischemic tissue is the most tried intervention. Transfer of a protein molecule is advantageous as it provides more controlled delivery, has established safety, and predictable pharmacokinetics. However, as these proteins degrade once they are injected into the body, they require sustained delivery. The alternative approach to overcome these drawbacks is to activate or inhibit specific genes through gene therapy. As hypoxia-inducible factors initiate an entire angiogenic response, they have been considered for angiogenic (gene) therapy.[34] However, caution is warranted as these hypoxia-inducible factors can also control cell death.[35] There are still many challenges before gene therapy can be applied to clinical settings. Gene expression can be manipulated by introducing therapeutic DNA, which must reach the target cells and transcribed. Transfer vectors are being investigated to deliver DNA to the cells.

## Challenges of Angiogenic Therapy

However, there are many challenges in administering these treatments in patients. VEGF forms leaky and tortuous vessels, which may be controlled by combining it with Ang1. Whether this abnormal vascular morphology can lead to impaired microcirculation is not known.[36] Furthermore, the adverse effects of increased levels of angiogenic factors such as triggering of dormant tumors and acceleration of atherosclerosis is not known. Another question is whether a single or a combination of angiogenic molecules will be required to stimulate ‘functional and sustainable’ angiogenesis. For example, genetic studies have shown that the VEGF120 isoform alone is able to initiate, but not complete, the angiogenic program.[37]

## Antiangiogenic Therapy

There are several diseases that will benefit from the inhibition of excessive angiogenesis [Table 2].[7] The approved antiangiogenic drugs for various indications are highlighted in Table 3.[3] Besides these, several other antiangiogenic agents are in various phases of clinical trials. In general, five strategies are being used as antiangiogenic therapies by the investigators:

**Table 3:** Approved antiangiogenic agents for various indications[3]

*Agent*	*Mechanism*	*Indication*
*Monoclonal antibody therapies*		
Bevacizumab	Humanized VEGF antibody	Metastatic colorectal cancer (mCRC),
		Non-small cell lung cancer (NSCLC),
		Advanced breast cancer,
		Neovascular (wet) Age-related macular
		degeneration
Cetuximab	Chimeric IgG1 EGFR antibody	mCRC, head and neck cancer
Panitumumab	Humanized IgG2 anti-EGFR antibody.	mCRC
Trastuzumab	Humanized IgG1 HER-2 antibody	Breast cancer
Ranizumab	Recombinant Fab fragment of anti-VGF antibody	Ocular neovascularization
*Tyrosine Kinase Inhibitors (TKIs)*		
Erlotinib	Small molecule TKI-EGFR	NSCLC, pancreatic cancer
Sorafenib	TKI of VEGFR-1, VEGFR-2, VEGFR-3, PDGFR-β, Raf-1	Advanced renal cell carcinoma (RCC), advanced hepatocellular carcinoma
Sunitinib	TKI of VEGFR-1, VEGFR-2, VEGFR-3, PDGFR-β, RET	Advanced RCC, GIST
*Other anticancer Angiogenic Agents*		
Temsirolimus	Small molecule inhibitor of mTOR (mammalian target of rapamycin), part of PI3 kinase / AKT pathway involved in tumor cell proliferation and angiogenesis	Advanced renal cell carcinoma
Everolimus	Small molecule inhibitor of mTOR	Advanced renal cell carcinoma
Bortezomib	Proteasome inhibitor-indirect antiangiogenic action	Multiple myeloma (MM), mantle cell lymphoma
Thalidomide	Immunomodulatory, anti-inflammatory, antiangiogenic	MM
Lenalidomide	Immunomodulatory, anti-inflammatory, antiangiogenic	Myelodysplastic Syndrome, MM
rhEndostatin (available only in China)	Endogenous angiogenesis inhibitor; blocks VEGF and down regulates MMP-2/9	NSCLC
*Approved agents have antiangiogenic properties dermatology*		
Alitretinoin	Topical retinoid-downregulation of VEGF	AIDS-related Kaposi's sarcoma
Imiquimod	Toll-Like Receptor 7 agonist-downregulation of FGF-2 and MMP-9	Benign genital warts
		Malignant skin cancers
Interferon α	Endogenous antiangiogenic cytokine	Hemangiomas, giant cell tumor
*Approved agent for use in ophthalmologic indication*		
Ranibizumab	Recombinant humanized IgG1 kappa VEGF-A antibody	Neovascular (wet) age-related macular degeneration.
Pegaptanib	Pegylated oligonucleotide (aptamer) - binds to extracellular VEGF.	Neovascular (wet) age-related macular degeneration.

Adapted from Understanding angiogenesis (document on the internet) -http://www.angio.org/ua.php[3]

- Inhibitors of activated EC
- Inhibitors of EC intracellular signaling
- Inhibitors of ECM remodeling
- Inhibitors of adhesion molecules
- Inhibitors of angiogenic mediators or their receptors

## 1. Inhibitors of Activated EC

Several natural inhibitors of angiogenesis have been described [Table 2]. TSP-1 is constitutively produced by normal cells and is the main physiological inhibitor of angiogenesis. TSP-1 is downregulated, while the angiogenic activity is increased, during tumorigenesis. Accordingly, mutation of the tumor suppressor *p53* gene results in the loss of TSP-1 production and a switch to the angiogenic phenotype. Decrease in angiogenesis and inhibition of tumor growth occurs with overexpression of TSP-1.[38] The other most promising antiangiogenic drugs, angiostatin and endostatin, are derived from the tumor cells themselves. Angiostatin suppresses the growth of a number of human tumors and their metastases, in mice.[39] Endostatin, a fragment of collagen, derived through elastase-mediated cleavage,[40] has been isolated from the media of hemangioendothelioma (EOMA) cells. Endostatin specifically suppresses endothelial cell proliferation and shows potent inhibitory activity against many tumor cell lines. In recent times, a potent antiangiogenic agent aaAT (antiangiogenic antithrombin) has been purified from small cell lung cancer.[41] TNP-470 (AGM-1470), a synthetic derivative of the antibiotic fumagillin, is the most studied angiogenic inhibitor. TNP-470 has been shown to inhibit type 2 methionine aminopeptidase, resulting in the abrogation of the processing of methionine, which may lead to the inactivation of unidentified proteins essential for EC growth.[42] TNP-470 is effective in the treatment of a wide variety of tumors. Thalidomide also has antiangiogenic activity. It demonstrates marked responses in patients with multiple myeloma, including those who relapsed after high-dose chemotherapy. The tubulin-binding drug Combretastatin A-4, exhibits a selective toxicity for proliferating EC by induction of apoptosis.

### Inhibitors of EC intracellular signaling

Some compounds like Genistein and Lavendustin A have the ability to block the activity of the angiogenic factors by inhibiting the protein tyrosine kinase, which is the second messenger system of these factors. Apart from this Ang-2 Inhibits Tie-2.

## 2. Inhibitors of ECM Remodeling

Metalloproteinase inhibitors (MMPI) are theoretically the most promising antiangiogenic agents. MMPI research has focused on synthetic, orally available inhibitors. Several MMPIs ranging from broad spectrum inhibitors, which block most of the MMPs, to selective inhibitors, which selectively inhibit a particular MMP, have been developed. Despite significant activity in the preclinical models, MMPIs failed to demonstrate a significant effect in the advanced stage clinical trials in most human malignancies. Batimastat was the first MMPI to be evaluated in humans, but the trials were suspended due to its low oral bioavailability. MMPIs that have entered clinical trials for an oncologic indication include prinomastat, BAY 12-9566, BMS-275291, marimastat, MMI270(B), and Metastat.

## 3. Inhibition of Cell Adhesion Molecules

α_v_β_3_ integrin, an adhesion receptor for ECM components is present selectively on activated endothelial cells, and therefore, is an attractive target for antiangiogenic therapy. Both the peptide antagonist of α_v_β_3_ and an anti-α_v_β_3_ monoclonal antibody, inhibit adhesion-dependent signal transduction by angiogenic factors, leading to apoptosis of the activated endothelium. These compounds have been found to inhibit angiogenesis. Currently the clinical potential of an anti-α_v_β_3_ antibody, Etaracizumab, is being evaluated in various phases of clinical trials, for many patients with late-stage cancer.[43]

## 4. Inhibitors of Angiogenic Mediators or their Receptors

Angiogenesis is very sensitive to small changes in factors such as VEGF and FGF-2. Strategies to inhibit the production or release of these growth factors or to interfere with their receptor interactions have been developed. Anti-VEGF antibodies, soluble VEGF receptors, or dominant negative VEGFR-2 have proven successful in the treatment of various conditions such as wet ocular neovascularization and many types of cancers. Synthetic low molecular weight inhibitors of tyrosine kinase receptors have been designed to interfere with growth factor receptor signaling. As tumor cells may depend on different cytokines, the inhibition of one single growth factor may cause only a partial control of tumor growth. This has led to the development of a nonselective tyrosine kinase inhibitor, which inhibits VEGF, FGF-2, and PDGF tyrosine kinase receptors. Various tyrosine kinase inhibitors have already been approved for various anticancer indications. Various exogenous heparin analogs, such as, suramin, are being developed for their capacity to inhibit FGF-2-induced angiogenesis.[7]

### Challenges of antiangiogenic therapy

The long-term side effects of many antiangiogenic therapies are not known. Interference with VEGF signaling may cause tumour-dependent toxicity. Although vessel count is considered as a successful prognostic factor, it does not accurately predict vascular function. Furthermore, many tumors do not ‘shrink’ during various antiangiogenic therapies. Thus, new imaging methods are needed to monitor vascular function and a therapeutic response in patients. Finally, the angiogenic response may depend on the individual genetic constitution; hence, the administration of agents should be based on the biology of the individual, to generate maximum therapeutic benefit.

## Conclusion

The study of angiogenesis is making a profound impact on the biological and medical world. The hope of being able to build new, functional, and durable blood vessels in ischemic tissues, or conversely, to prevent their further growth in malignant and inflamed tissues is becoming more realistic every day. However, efforts to therapeutically stimulate new blood vessels have significantly lagged behind those to inhibit angiogenesis. Better understanding of the underlying process will enable the scientist to develop new drugs and therapies that will significantly enhance our ability to treat intractable diseases, such as, cancer, diabetes, and heart disease.
